# *Rickettsia* in mosquitoes, Yangzhou, China

**DOI:** 10.1038/emi.2016.107

**Published:** 2016-10-12

**Authors:** Jilei Zhang, Patrick John Kelly, Guangwu Lu, Luis Cruz-Martinez, Chengming Wang

**Affiliations:** 1 Jiangsu Co-innovation Center for Prevention and Control of Important Animal Infectious Diseases and Zoonoses, Yangzhou University College of Veterinary Medicine, Yangzhou 225009, Jiangsu Province, China; 2 Department of Clinical Sciences, Ross University School of Veterinary Medicine, Basseterre 00265, St. Kitts and Nevis, West Indies; 3 Department of Pathobiology, College of Veterinary Medicine, Auburn University, Auburn, AL 36849, USA

**Dear Editor,**

Spotted fever group (SFG) rickettsiae are obligate intracellular Gram-negative bacteria that are most commonly transmitted by a range of ticks and fleas. Recently, attention has become focused on mosquitoes as vectors of SFG rickettsiae with *Rickettsia felis* being identified by PCR in *Aedes* spp., *Anopheles* spp. and *Mansonia uniformis* in Africa,^1, 2, 3^ and *Anopheles sinensis* and *Culex pipiens* in China.^4^ Further, it has been shown that *Anopheles gambiae* can be infected with *R. felis* and transmit the organism during feeding, indicating it might be a potential vector.^5^ Finally, a new *Rickettsia* species has been found in *An. gambiae* and *Anopheles melas* in Africa,^3^ the blood of a patient from Senegal,^3^ and in *Cx. pipiens* in China.^4^ To gather more data on *Rickettsia* species in mosquitoes in China, we studied convenience samples of mosquitoes from Yangzhou and report our findings below.

In June 2015, convenience samples of mosquitoes were captured with hand nets in the environs of the halls of residence of Yangzhou University. The species and gender of the trapped mosquitoes were determined using standard morphological criteria (www.wrbu.org/morph_MQ.html) before the *Cx. pipiens* males and females and the *Aedes albopictus* were pooled separately (15 per pool) in 600 μL of RNA/DNA Stabilization Reagent for Blood/Bone Marrow (Roche Molecular Biochemicals, Indianapolis, IN, USA) and stored at −80 °C. The DNA was extracted from the pooled mosquitoes as described before^4^ and tested for *Rickettsia* with a *gltA*-based FRET-PCR and a nested-PCR as described before.^4^ To further characterize the *Rickettsia* found in the positive mosquito pools, we used multilocus sequence typing (MLST) with primers against the *gltA* which we developed (forward primer: 5'-ATG AGC AGA ATG CTT CTA CTT CAA CA-3'; reverse primer: 5'-ATT TTC TCT CAA TAA AAT ATT CAT CTT TAA G-3'), and those previously described for *ompA*, *ompB* and *sca4*.^3, 6, 7^ A FRET-PCR targeting the mammalian hydroxymethylbilane synthase (HMBS) gene^8^ and a regular PCR we designed to amplify the avian HMBS gene (forward primer: 5'-TTA GCA GTG GAA GTT CGT GCC AA-3' reverse primer: 5'-AGG GAC ACT ACA GCC ACC CTC CT-3') were used to determine whether mosquitoes had ingested a blood meal.

In total, we captured 450 male and 345 female *Cx. pipiens* and found 30% (9/30) of the male pools and 9% (2/23) of the female pools positive by PCR for *Rickettsia* species. None of the male pools were positive by PCR for the HMBS, which was expected as males only feed from plants. The two female pools positive for *Rickettsia* species were negative for the HMBS suggesting that infected females were unfed. Ten of the remaining 21 female pools were positive for the mammalian and/or avian HMBS genes showing blood meals had been taken from mammals (*n*=2), birds (8) and both (3).

The MLST we performed showed the *Rickettsia* species in all 11 of our mosquito pools were identical and most closely related to the new *Rickettsia* species (JN620082/JQ354961) in *An. gambiae* and *An. melas* from Cote d'Ivoire, Africa,^3^ and a *Rickettsia* sp. (JQ674485) in blood from a patient in Senegal^3^ (Figure 1). It was more distantly related to *R. felis* from cat fleas (*Ctenocephalides felis*; HM582437, CP000053, NC_007109, DQ408668), mosquitoes (*An. melas*; JQ674484),^1^ people (KP318094) and book lice (*Liposcelis bostrychophila*; GQ329878).

None of the male (10) or female (14) pools of *Ae. albopictus* we collected were positive for *Rickettsia* species with some of the female pools (29% 4/14) having evidence of a mammalian and/or avian blood meal.

Our study adds to the growing evidence that mosquitoes might have a role in the epidemiology of infections with SFG rickettsiae. Since the first reports that rickettsiae could be propagated in mosquito cell lines,^9, 10^ there has been growing evidence that mosquitoes can be infected with the organisms.^1, 2, 3, 4, 5^ Our study provides further information on *Rickettsia* species in mosquitoes, in particular the common house mosquito *Cx. pipiens* that occurs widely in temperate areas of the world and feeds most commonly on a wide variety of bird species but also occasionally on people and a wide range of mammals.^11^ We found relatively large numbers of pools of *Cx. pipiens* contained *Rickettsia* (11/53; 21%) indicating infections are common in this species. This is supported by unpublished data from our laboratory on individual *Cx. pipiens* which shows around 8% of females and 1% of males are infected. MLST indicated the *Rickettsia* in our pooled samples was the new *Rickettsia* species reported in *An. gambiae* and *An. melas* in Africa, or a very closely related species. As noted previously, the definitive characterization of this species will depend on isolates becoming available, which will enable more thorough analysis.^3^

In a previous study,^4^ we found DNA of *R. felis* in 5% of the *Cx. pipiens* we studied but all were negative in the current study. We were not able to determine a reason for this difference but it is known that numbers of mosquitoes infected with *Rickettsia*^2^ and dengue virus^12^ can vary with season and this might have been the case with our samples which were collected in spring (April to June) in the current study but in autumn (October and November) in the previously published study.

It is of note that we found the new *Rickettsia* species or a closely related organism in relatively large numbers of the pools of male *Cx. pipiens*. Male mosquitoes do not take blood meals, as confirmed by our negative mammalian and avian HMBS gene PCRs, and hence the organisms were not part of a blood meal ingested from a rickettsemic host.

Such vertical transmission of SFG rickettsiae is not unusual and has been demonstrated in ticks, *R. africae* in *Amblyomma hebraeum* for example,^13^ and in fleas, *R. felis* in *C. felis* for example.^14^ Studies on transmission in mosquitoes have been equivocal with vertical transmission not observed in *An. gambiae* experimentally infected with *R. felis* despite organisms being present in the ovaries.^5^ On the other hand, *R. felis* was found in a male *An. arabiensis*, which suggested vertical transmission.^2^

Our study shows a new *Rickettsia* species first described in *An. gambiae* and *An. melas* in Africa, or a closely related species, appears to also occur in China in *Cx. pipiens*. Detection of the *Rickettsia* in pools of male *Cx. pipiens* and unfed female *Cx. pipiens* suggests there might be transovarial transmission. Further studies are needed to characterize the organism, its transmission and its potential role as a pathogen, particularly as it might infect people.^3^

## Figures and Tables

**Figure 1:**
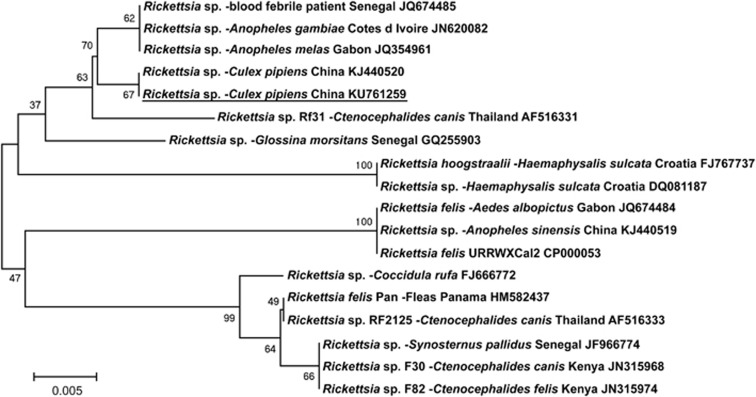
Phylogenetic tree using a bootstrap analysis for the *Rickettsia* species in *Culex pipiens* from China. The nearest GenBank sequences (shown at the end of the sequence name) were aligned using the multi-sequence alignment program. The evolutionary tree was constructed using parsimony and maximum-likelihood methods. The *Rickettsia* sp. found in this study (underlined) was identical to the sequence we studied before, and was closest to the new *Rickettsia* species in *An. gambiae* and *An. melas* from Africa.
